# Development of a clinical prediction model for inflammatory biomarkers and enlarged basal ganglia perivascular spaces using SHAP analysis: feature selection and model interpretation

**DOI:** 10.3389/fneur.2025.1665841

**Published:** 2026-01-02

**Authors:** Jiayu Lv, Dewang Gao, Wen Yong, Wenlong Yu, Lu Wang, Shangjia Ma, Hua Li, Shuaiqiang Zhang, Zi Guo, Hao Yan, Zhipeng Ju, Yiming Liu, Lie Wu, Xia Guo

**Affiliations:** 1 Department of Neurology, The First Affiliated Hospital of Baotou Medical College, Baotou, China; 2 Baogang Hospital of Inner Mongolia, Baotou, China

**Keywords:** enlarged perivascular spaces (EPVS), systemic inflammation response index (SIRI), Montreal Cognitive Assessment (MoCA), SHapley Additive exPlanations (SHAP), clinical prediction model

## Abstract

**Background:**

Enlarged perivascular spaces (EPVS) may lead to dysfunction of the cerebral lymphatic system, which may be associated with cerebrovascular diseases, cognitive dysfunction, and other neurological diseases. However, the association between cognitive function and systemic inflammation has not been systematically elucidated. This study aimed to develop a predictive model integrating the Montreal Cognitive Assessment (MoCA) and complete blood count-derived inflammatory markers to analyze the relationship between multidimensional indicators and BG-EPVS burden.

**Methods:**

We consecutively enrolled patients with cerebral small vessel disease (CSVD) admitted to the Department of Neurology, First Affiliated Hospital of Baotou Medical College, between 2023 and 2024. BG-EPVS severity was evaluated using MRI, and statistical analyses were conducted on clinical variables. Univariate and multivariate logistic regression analyses were conducted to identify independent predictors of BG-EPVS severity. Model performance and clinical utility were evaluated using receiver operating characteristic curves (ROC-AUC), calibration plots, decision curve analysis (DCA), and clinical impact curves (CIC). Model interpretability was assessed using SHapley Additive exPlanations (SHAP).

**Results:**

Multivariate logistic regression identified MoCA score, age, hypertension, SIRI and education independent predictors of BG-EPVS burden.

**Conclusions:**

These findings demonstrate that age, hypertension and SIRI were positively correlated with high BG-EPVS burden, while MoCA score and education duration were negatively correlated. The integrated model combining MoCA and inflammatory biomarkers accurately predicts BG-EPVS burden, demonstrating their clinical value in early disease screening and dynamic monitoring.

## 1 Introduction

Enlarged perivascular spaces (EPVS) is a pathological condition characterized by the dilation of fluid-filled spaces around intracranial veins, small veins, arteries, and small arteries, it may reflect a failure of interstitial fluid circulation owing to disruption of the blood brain barrier in small vessels (1). A recent study (2) indicates that EPVS predominantly occurs in individuals aged 35 and above. Studies by Rollins et al. (3) and Groeschel et al. (4) reported EPVS prevalence rates among children and healthy adults is only 1.6–3%. As people age, the incidence of EPVS rises in tandem, resulting in corresponding socioeconomic burdens. When PVS expands to a certain extent, it can be detected by MRI as EPVS. PVS with a diameter ≤ 2 mm is considered a normal anatomical structure, while those exceeding 2 mm are termed EPVS (5). Recent studies indicate that EPVS may be closely associated with recurrent ischemic stroke, poor prognosis of cerebral hemorrhage, cognitive impairment, and cerebral amyloid angiopathy (6–9). This may be because intracranial lesions trigger inflammatory signals, inducing a series of severe reactions that lead to cell apoptosis and subsequently cause brain damage (7). Furthermore, this may also be associated with lymphatic return obstruction and blood-brain barrier disruption (10). Although the exact mechanism is not clear, inflammation plays an important role.

Chronic inflammation is a low-grade, non-contagious, systemic inflammatory state that is closely associated with aging, psychological factors, environmental influences, lifestyle, and the resolution of acute inflammation (11). This inflammatory state may lead to a series of pathological processes such as perivascular cell injury and blood-brain barrier dysfunction, which in turn alter cerebrospinal fluid dynamics and metabolite clearance (12). As the core mechanism of EPVS, chronic inflammation plays a full role in the development of this pathological condition. In recent years, inflammatory markers based on complete blood count, especially composite biomarkers calculated from the count of neutrophils, lymphocytes and platelets, have been widely used in clinical practice. These indicators are also called new inflammatory markers (13). Multiple studies have repeatedly confirmed that these novel inflammatory markers have potential value in the early prediction and prognosis assessment of cerebrovascular diseases (14–17). However, the relationship between these readily available inflammatory markers and EPVS development remains unclear, requiring further research to confirm. Therefore, this study aims to investigate the correlation between novel inflammatory markers and EPVS to identify better clinical early warning indicators. These findings in community hospitals help better predict the occurrence of EPVS early, aiming to achieve early diagnosis, early intervention, and early treatment, thereby improving patients' quality of life.

## 2 Materials and methods

### 2.1 Study design and participants

We retrospectively included 327 patients CSVD who were admitted to the Department of Neurology at the First Affiliated Hospital of Baotou Medical College between December 2023 and December 2024. Inclusion criteria: (1) for patients admitted with the diagnostic criteria for CSVD (5): upon medical imaging examination, patients exhibit at least one of the following four imaging markers: presumed vascular origin lacunes, WMH, PVS, and CMBs; (2) age ≥40 years; (3) Complete laboratory and imaging data. Exclusion criteria: (1) hematological disorders or acute infectious diseases; (2) a history of acute cerebral infarction (within 14 days), acute cerebral hemorrhage, aneurysms, or arteriovenous malformations; (3) demyelinating disorders (e.g., multiple sclerosis, neuromyelitis optica); (4) epilepsy, malignant tumors, or severe cardiopulmonary, hepatic, or renal dysfunction; (5) inability to complete cognitive assessments. The study protocol was approved by the Ethics Committee of First Affiliated Hospital of Baotou Medical College (Approval No.: 2024-K044-01). All procedures were conducted in strict accordance with ethical guidelines, and we applied for exemption from written informed consent.

### 2.2 Data collection

Demographic data (age, sex), medical history (e.g., diabetes mellitus, hypertension), and lifestyle factors (e.g., smoking, alcohol consumption) were collected for each patient. Upon admission, laboratory data were collected, including a complete blood count (with parameters for calculating SIRI), fasting blood glucose, lipid profile (total cholesterol, triglycerides, LDL-C, HDL-C), liver, and renal function tests. Data collection was performed by trained neurologists at First Affiliated Hospital of Baotou Medical College, with strict privacy protection measures implemented throughout the process to ensure the confidentiality of participant information.

### 2.3 Laboratory testing

Venous blood samples were collected after overnight fasting. Complete blood count analysis was performed using automated hematology analyzers. Calculated inflammatory biomarkers included: NLR (neutrophil count/lymphocyte count); dNLR [neutrophil count/(white blood cell count – neutrophil count)]; LMR (lymphocyte count/monocyte count); PLR (platelet count/lymphocyte count); SII (neutrophil count × platelet count/lymphocyte count); LWR (lymphocyte count/leukocyte count); NWR (neutrophil count/leukocyte count); PNR (platelet count/neutrophil count); SIRI (neutrophil count × monocyte count/lymphocyte count); and ELR (eosinophil count/lymphocyte count).

### 2.4 Cognitive assessment

MoCA evaluated eight cognitive domains: visuospatial/executive function, naming, memory, attention, language, abstraction, delayed recall, and orientation. Total score: 30 points; ≥26 indicated normal cognition. One point was added for individuals with ≤ 12 years of education. Assessments were completed within 3 days of enrollment by trained neuropsychologists.

### 2.5 Neuroimaging

Within 7 days of admission, patients underwent 3.0T MRI (Siemens Healthineers, Germany) with sequences including T1-weighted imaging (T1WI), T2-weighted imaging (T2WI), diffusion-weighted imaging (DWI), fluid-attenuated inversion recovery (FLAIR), and susceptibility-weighted imaging (SWI). T2WI was the primary sequence for evaluating EPVS, while T1WI and FLAIR were used as reference sequences. DWI was used to evaluate acute cerebral infarction, and magnetic resonance angiography (MRA) assessed intracranial and cervical vasculature. Without knowledge of the patient's basic information, two senior neuroimaging specialists, who had passed the consistency test, visually reviewed the MRI images according to the “Chinese Consensus on the Diagnosis and Treatment of Cerebral Small Vessel Diseases.” Discrepancies were resolved through consensus review. EPVS demonstrate cerebrospinal fluid-isointense signals: hypointense on T1WI and FLAIR (without perivascular hyperintensity on FLAIR), and round/ovoid or linear hyperintensity on T2WI. Severity was graded per Potter et al. (18): grade 0 (absent), 1 ( ≤ 10), 2 (11–20), 3 (21–40), and 4 (>40).

**Figure 1:**
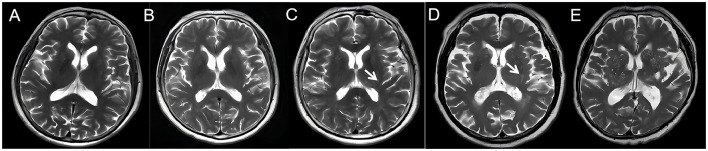
Axial T2-weighted MRI of the brain demonstrating BG-EPVS at different severity grades (0–4). **(A)** Grade 0 **(B)** grade 1 **(C)** grade 2 **(D)** grade 3 **(E)** grade 4. The white arrow points to EPVS.

BG-EPVS quantification involved counting EPVS on the single most affected unilateral slice on T2WI. Subjects were stratified into two groups: mild (grades 0–1, ≤ 10 EPVS) and moderate-to-severe (grades 2–4, >10 EPVS; Figure 1).

### 2.6 Model construction

**Figure 2:**
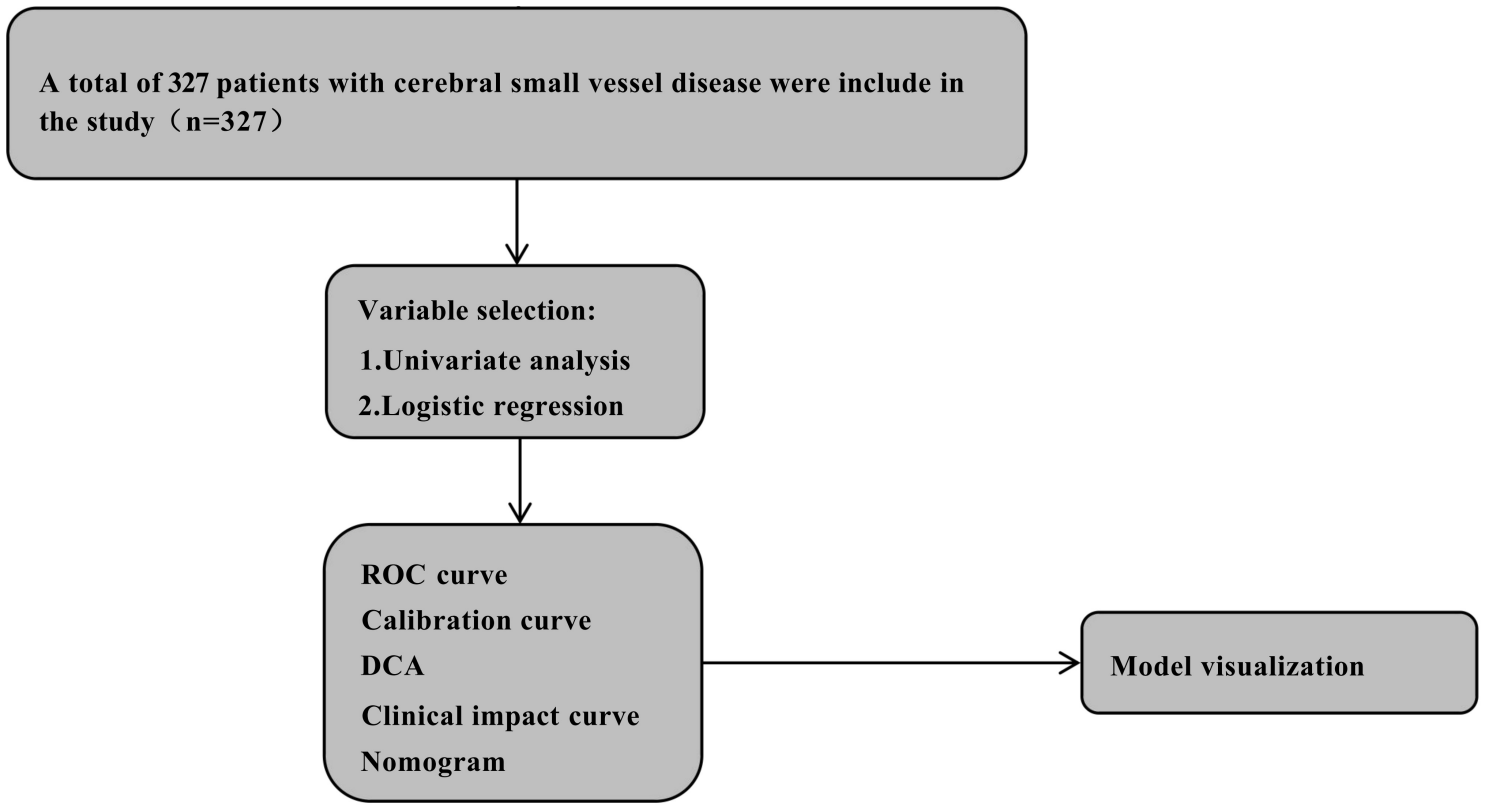
The flow chart of this study. CSVD, cerebral small vessel disease; ROC, receiver operator characteristic; DCA, decision curve analysis.

Univariate and multivariate logistic regression analysis identified clinical variables, with variance inflation factors < 5 indicating no significant multicollinearity. The prediction model's performance was validated using: (1) receiver operating characteristic curves (ROC-AUC): used to evaluate the model's ability to distinguish between severe and non-severe BG-EPVS cases, with values closer to 1 indicating better discrimination. (2) Calibration curves: used to evaluate the consistency between the predicted probability of the model and the observed probability of the actual model. The ideal calibration curve should be close to the diagonal. (3) Decision curve analysis (DCA): used to evaluate the clinical net benefit of the model at different decision thresholds to help determine the clinical utility of the model. (4) Clinical impact curves (CIC): demonstrates the clinical impact of predictive models across different risk thresholds, helping researchers assess the model's real-world applicability by evaluating how well its predictions align with actual clinical outcomes. A closer alignment between the red and blue curves indicates higher accuracy, as they correspond more closely to the actual prevalence of high-risk populations. The Shapley Additive Explanations (Shap) method, a game-theoretic approach for explaining machine learning outputs, calculates each feature's contribution to individual predictions, thereby assessing feature importance. Model interpretability was demonstrated via SHAP summary plots and beeswarm visualizations. The overall study design is presented in Figure 2.

### 2.7 Statistical analysis

SPSS 27.0 and R 4.2.2 were used. Normally distributed data: mean ± SD (independent *t*-tests); for non-normally distributed measurement and ordinal data, the median and interquartile range [M (P25, P75)] were used, and the Mann–Whitney *U* test was applied. Spearman correlation was used to analyze relationships between inflammatory and imaging markers and cognition. A binary logistic regression model was used to identify risk factors, adjust for confounding variables such as gender, history of cerebrovascular disease, hypertension, and diabetes, and explore the interaction effects and subgroup analysis of SIRI. The ROC curve was used to analyze the cut-off value, sensitivity, and specificity of relevant indicators in the groups, predict EPVS values, and generate the nomogram. A *P*-value of < 0.05 was considered statistically significant. R packages: pROC, CBCgrps, rms, and rmda.

## 3 Results

### 3.1 Baseline characteristics

A total of 327 eligible participants were included in this study. The final cohort consisted of 188 participants with mild BG-EPVS and 139 with moderate-to-severe BG-EPVS. No significant differences were observed between the groups in terms of sex, diabetes, LDL, HDL, fasting blood glucose, TG, CR, UA, PLR, ELR, and SII (*P*>0.05). However, significant differences were found in age, hypertension, smoking and education (*P* < 0.05). The moderate-to-severe group exhibited lower levels of LMR, PMR, LWR, PNR, education and Moca, but higher levels of TC, NLR, SIRI, Dnlr, and NWR compared to the mild group (Table 1).

**Table 1:** Baseline characteristic of the study subject.

**Variables**	**Total (*n* = 327)**	**Mild (*n* = 188)**	**Moderate-severe (*n* = 139)**	**Statistic**	***P***
Age, years	66.18 ± 9.17	63.88 ± 9.23	69.29 ± 8.14	*T* = 5.51	< 0.001
≤ 65	158 (48.32)	112 (59.57)	46 (33.09)	*χ^2^* = 22.44	< 0.001
>65	169 (51.68)	76 (40.43)	93 (66.91)		
Male, *n* (%)	165 (50.46)	100 (53.19)	65 (46.76)	χ^2^ = 1.32	0.250
Smoking, *n* (%)	251 (76.76)	154 (81.91)	97 (69.78)	*χ^2^* = 6.59	0.010
Alcohol history, *n* (%)	284 (86.85)	170 (90.43)	114 (82.01)	*χ^2^* = 4.95	0.026
Hypertension, *n* (%)	126 (38.53)	93 (49.47)	33 (23.74)	*χ^2^* = 22.33	< 0.001
Diabetes, *n* (%)	246 (75.23)	147 (78.19)	99 (71.22)	*χ^2^* = 2.08	0.149
Neutrophils, × 10^9^/L	4.26 (3.23, 5.37)	4.02 (3.06, 5.14)	4.41 (3.58, 5.50)	*Z* = −2.10	0.036
Lymphocytes, × 10^9^/L	1.54 (1.20, 2.00)	1.64 (1.25, 2.00)	1.45 (1.12, 1.96)	*Z* = −1.88	0.060
Platelets, × 10^9^/L	212.00 (171.50, 251.00)	214.50 (177.00, 256.00)	206.00 (165.50, 248.50)	*Z* = −1.51	0.131
Monocyte, × 10^9^/L	0.43 (0.34, 0.54)	0.40 (0.33, 0.53)	0.47 (0.35, 0.58)	*Z* = −2.69	0.007
Eosinophils, × 10^9^/L	0.07 (0.03, 0.14)	0.07 (0.03, 0.14)	0.07 (0.04, 0.13)	*Z* = −0.02	0.987
Basophils, × 10^9^/L	0.03 (0.02, 0.04)	0.03 (0.02, 0.04)	0.03 (0.02, 0.04)	*Z* = −0.36	0.716
WBC, × 10^9^/L	6.55 (5.50, 7.76)	6.36 (5.41, 7.59)	6.71 (5.66, 7.97)	*Z* = −1.63	0.104
HGB, g/L	144.02 ± 17.25	144.13 ± 14.88	143.88 ± 20.07	*t* = 0.12	0.902
Metabolic syndrome, *n* (%)	117 (35.78)	55 (29.26)	62 (44.60)	*χ^2^* = 8.19	0.004
LDL, mmol/L	2.56 (2.08, 3.19)	2.66 (2.14, 3.25)	2.50 (1.94, 3.06)	*Z* = −1.77	0.077
HDL, mmol/L	1.11 (0.94, 1.31)	1.12 (0.96, 1.31)	1.08 (0.92, 1.25)	*Z* = −1.53	0.127
FPG, mmol/L	5.50 (4.90, 6.40)	5.30 (4.90, 6.15)	5.60 (5.00, 6.65)	*Z* = −1.83	0.068
TG, mmol/L	1.44 (0.99, 2.02)	1.48 (1.05, 2.10)	1.37 (0.92, 1.90)	*Z* = −1.64	0.101
TC, mmol/L	4.17 (3.52, 4.97)	4.37 (3.68, 5.05)	3.97 (3.32, 4.87)	*Z* = −2.48	0.013
CR, μmol/L	70.00 (59.00, 84.00)	68.00 (58.75, 80.00)	72.00 (60.50, 86.50)	*Z* = −1.91	0.056
UA, μmol/L	323.00 (267.00, 387.50)	322.50 (267.50, 386.75)	327.00 (265.50, 387.00)	*Z* = −0.02	0.982
NLR	2.62 (1.89, 3.73)	2.42 (1.82, 3.51)	2.85 (2.09, 3.92)	*Z* = −2.79	0.005
LMR	3.64 (2.68, 4.88)	4.03 (2.95, 5.01)	3.31 (2.49, 4.64)	*Z* = −3.48	< 0.001
PMR	485.71 (357.57, 661.80)	519.54 (373.83, 704.63)	444.23 (335.90, 561.65)	*Z* = −3.22	0.001
LWR	0.25 (0.19, 0.31)	0.26 (0.20, 0.32)	0.23 (0.19, 0.29)	*Z* = −3.00	0.003
PNR	50.87 (37.39, 64.47)	53.55 (41.01, 66.97)	46.79 (36.55, 59.73)	*Z* = −2.77	0.006
SIRI	1.15 (0.73, 1.76)	1.00 (0.67, 1.50)	1.35 (0.82, 1.89)	*Z* = −3.53	< 0.001
Dnlr	0.65 (0.59, 0.72)	0.64 (0.58, 0.72)	0.67 (0.60, 0.73)	*Z* = −2.17	0.030
PLR	133.06 (104.68, 173.14)	130.60 (104.62, 172.33)	134.20 (106.40, 174.92)	*Z* = −0.51	0.608
NWR	0.65 (0.59, 0.72)	0.64 (0.58, 0.72)	0.67 (0.60, 0.73)	*Z* = −2.17	0.030
ELR	0.05 (0.02, 0.08)	0.04 (0.02, 0.08)	0.05 (0.02, 0.08)	*Z* = −1.06	0.290
SII	533.43 (377.90, 815.03)	504.87 (361.06, 780.74)	583.09 (415.68, 864.67)	*Z* = −1.72	0.085
Education, *n* (%)				*χ^2^* = 39.67	< 0.001
Less than high school education	212 (64.83)	95 (50.53)	117 (84.17)		
High school degree or above	115 (35.17)	93 (49.47)	22 (15.83)		
MoCA	22.00 (15.00, 27.00)	24.00 (20.75, 27.00)	16.00 (11.00, 22.00)	*Z* = −7.91	< 0.001

Values for continuous variables are expressed as mean ± standard deviation or interquartile range; values for categorical data are given as numbers (percent). BG-EPVS mild group (grade 0–1: ≤ 10). BG-EPVS moderate and severe group (grade 2–4: >10). NLR, neutrophil count/lymphocyte count; dNLR, neutrophil count/(white blood cell count – neutrophil count); LMR, lymphocyte count/monocyte count; PLR, platelet count/lymphocyte count; SII, neutrophil count × platelet count/lymphocyte count; LWR, lymphocyte count/white blood cell count; NWR, neutrophil count/white blood cell count; PNR, platelet count/neutrophil count; SIRI, (neutrophil count × monocyte count)/lymphocyte count; ELR, eosinophil count/lymphocyte count; *P* values are compared between mild and moderate-severe groups. *t, t*-test; *Z*, Mann-Whitney test; χ^2^, Chi-square test.

### 3.2 Feature selection

Thirty-five variables were analyzed for differences, including demographic characteristics, metabolic indicators, and systemic inflammatory markers. Significant differences between the two groups were found in age, Smoking, Alcohol history, Metabolic syndrome, Hypertension, Education, Neutrophils, Monocyte, TC, NLR, LMR, PMR, LWR, PNR, SIRI, dNLR, NWR, and MoCA score.

**Figure 3:**
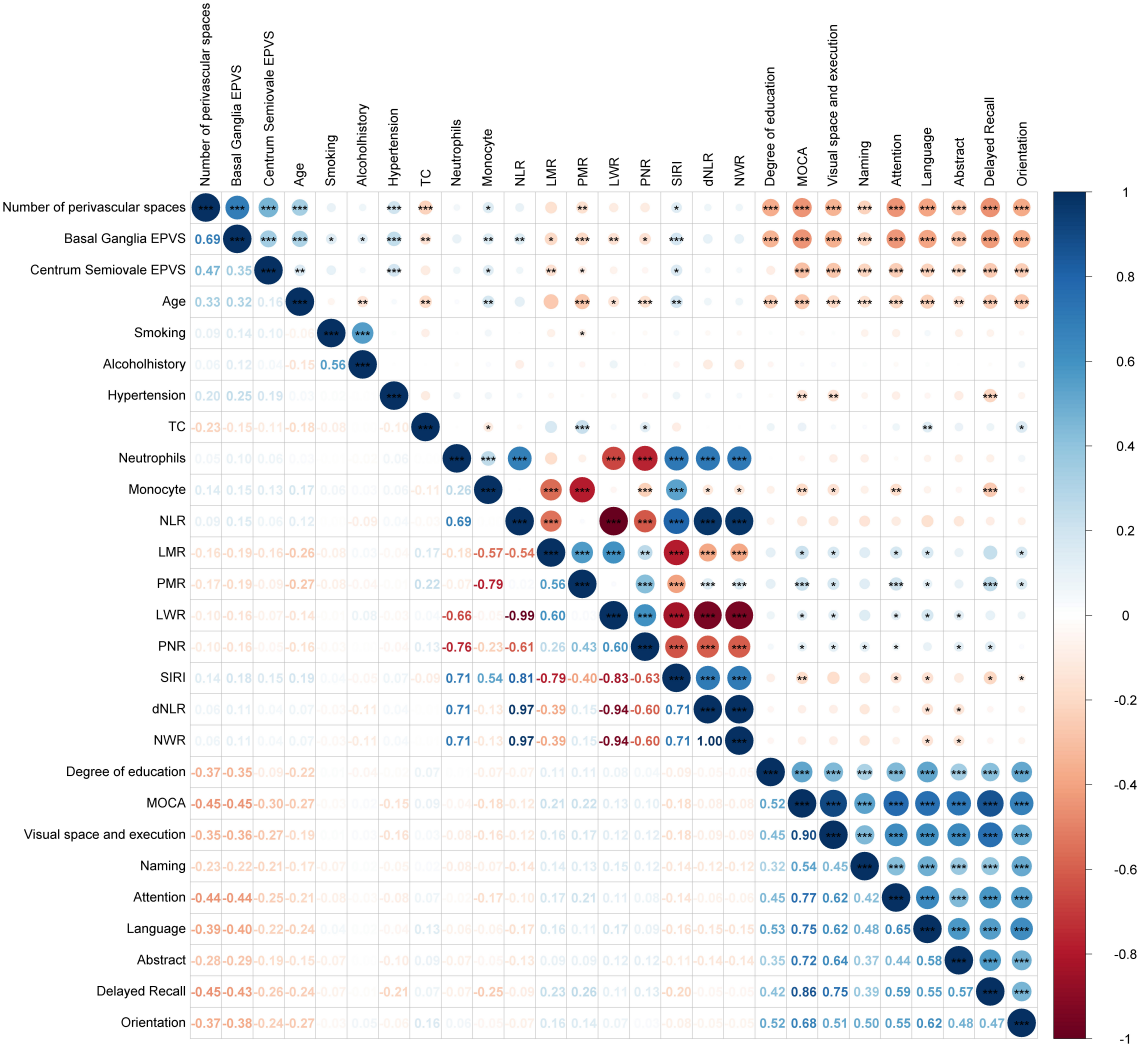
Correlation heat map of EPVS variables. The upper triangle represents the significant relationship between the variables, the lower triangle shows the correlation coefficient, the circle size represents the size of the correlation coefficient, color depth represents correlation coefficient (*r* value), ^*^ <0.05; ^**^ <0.01; ^***^ <0.001.

Correlation analysis will be performed on 27 variables with significant differences, 15 of which are correlated. Significant correlations were found between BG-EPVS and age (*r* = 0.33, *P* < 0.001), hypertension (*r* = 0.20, *P* < 0.001), SIRI (*r* = 0.14, *P* < 0.05), education (*r* = −0.37, *P* < 0.001), and MoCA (*r* = −0.45, *P* < 0.001) (Figure 3).

**Figure 4:**
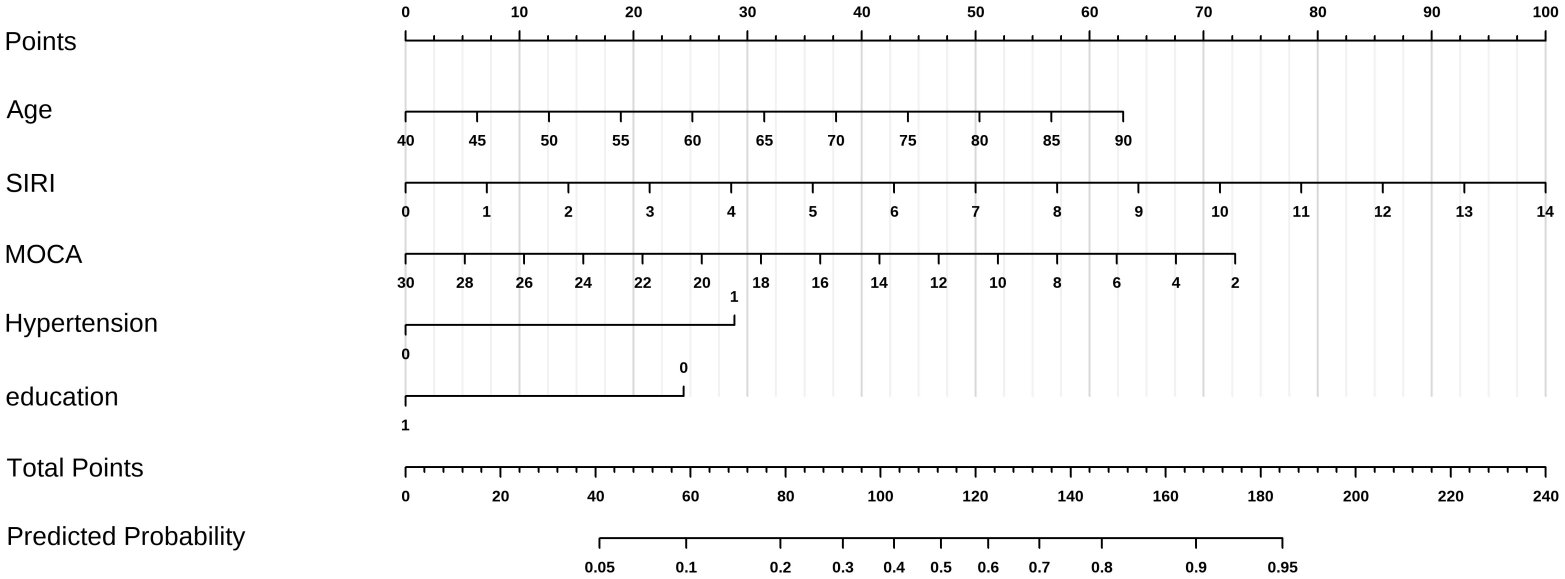
A nomogram for BG-EPVS burden. The vertical line at the top of the column chart is used to obtain the total score (0–100 points) for each independent variable. The total scores of all variables are added together to obtain the forecast risk value on the prediction line at the bottom of the column chart. Distribution of categorical variables, without disease or state, represented by no, otherwise represented by yes. In the two categorical variables of hypertension and Lac score, 0 is no and 1 is yes.

Variables with significance (*P* < 0.1) in univariate analysis were entered into multivariate backward stepwise logistic regression. Age, hypertension, SIRI, education, and MoCA score were identified as independent risk factors for BG-EPVS burden (Table 2). Convert the age group variable in the model to numeric. Subsequently, a nomogram was constructed and the predictive model was evaluated (Figure 4).

**Table 2:** Single and multiple factor logistic regression analysis.

**Variables**	**Single factor**					**Multiple factor**				
	β	**S.E**	***Z***	***P***	**OR (95% CI)**	β	**S.E**	***Z***	***P***	**OR (95% CI)**
Age										
42–58					1.00 (Reference)					1.00 (Reference)
59–75	1.46	0.43	3.37	< 0.001	4.32 (1.84–10.14)	1.28	0.49	2.62	0.009	3.59 (1.38–9.34)
76–92	1.82	0.47	3.88	< 0.001	6.16 (2.46–15.45)	1.30	0.54	2.41	0.016	3.67 (1.28–10.54)
Hypertension	1.15	0.25	4.64	< 0.001	3.14 (1.94–5.10)	1.20	0.29	4.13	< 0.001	3.31 (1.88–5.85)
TC	−0.25	0.11	−2.29	0.022	0.78 (0.63–0.96)					
PMR	−0.01	0.00	−2.89	0.004	0.99 (0.99–0.99)					
SIRI	0.38	0.12	3.13	0.002	1.46 (1.15–1.86)	0.32	0.13	2.40	0.016	1.38 (1.06–1.79)
MOCA	−0.16	0.02	−7.52	< 0.001	0.85 (0.82–0.89)	−0.11	0.02	−4.59	< 0.001	0.90 (0.86–0.94)
Education	−1.65	0.27	−6.01	< 0.001	0.19 (0.11–0.33)	−1.04	0.33	−3.15	0.002	0.35 (0.19–0.68)

OR, odds ratio; CI, confidence interval.

### 3.3 Model performance

**Figure 5:**
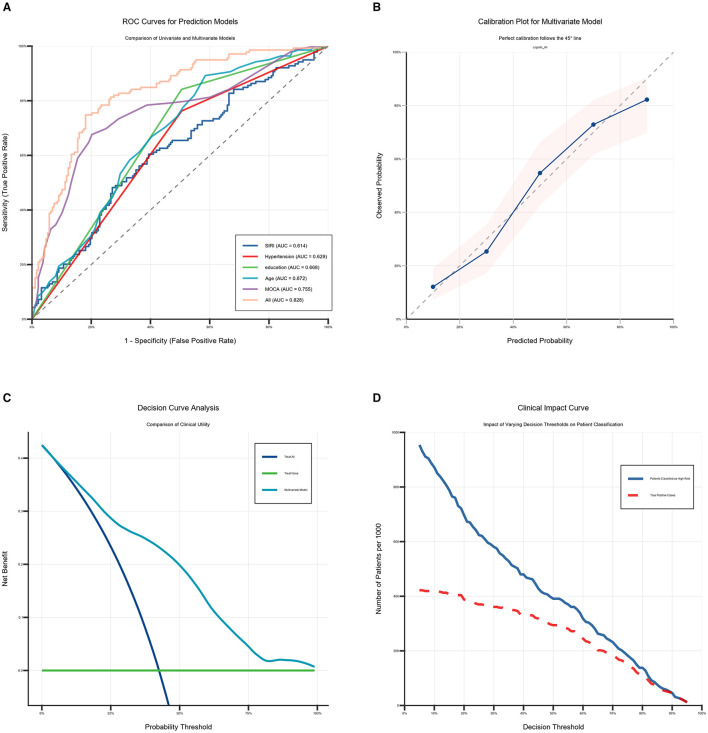
Five variables combined to predict performance. **(A)** The receiver operating characteristic curve (ROCauc) curve five variables combined to predict performance. **(B)** The calibration curve of five variables combined to predict performance. **(C)** The decision curve analysis (DCA) of five variables combined to predict performance. **(D)** The clinical impact curve (CIC) of five variables combined to predict performance.

ROC analysis: SIRI predicted moderate-severe BG-EPVS: AUC = 0.614 (95% CI: 0.553–0.676; *P* < 0.001); cutoff: 1.3993 (sensitivity: 48.2%; specificity: 72.9%). MoCA predicted moderate-severe BG-EPVS: AUC = 0.755 (95% CI: 0.700–0.810; *P* < 0.001); cutoff: 19.5 (sensitivity: 79.8%; specificity: 67.6%). Combined predicted moderate-severe BG-EPVS: AUC = 0.828 (95% CI: 0.783–0.873; *P* < 0.001); cutoff: 0.457 (sensitivity: 74.8%; specificity: 81.9%). Combined prediction outperformed single markers (Figure 5A). We evaluated the model's accuracy in predicting the probability of BG-EPVS in CSVD patients by analyzing the calibration curve, DCA, and CIC. The calibration curve of the prediction model indicates consistency between the model's predictions and actual incidence (Figure 5B). DCA demonstrated greater net benefit than “treat-none” or “treat-all” strategies at threshold probabilities 0.03–1.0 (Figure 5C). CIC confirmed clinical utility across practical threshold probabilities (Figure 5D). The CIC clearly demonstrates that the model provides superior overall net benefit across a broad range of threshold probabilities and influences patient outcomes, highlighting the significant predictive value of the combined prediction model.

### 3.4 Model interpretability

**Figure 6:**
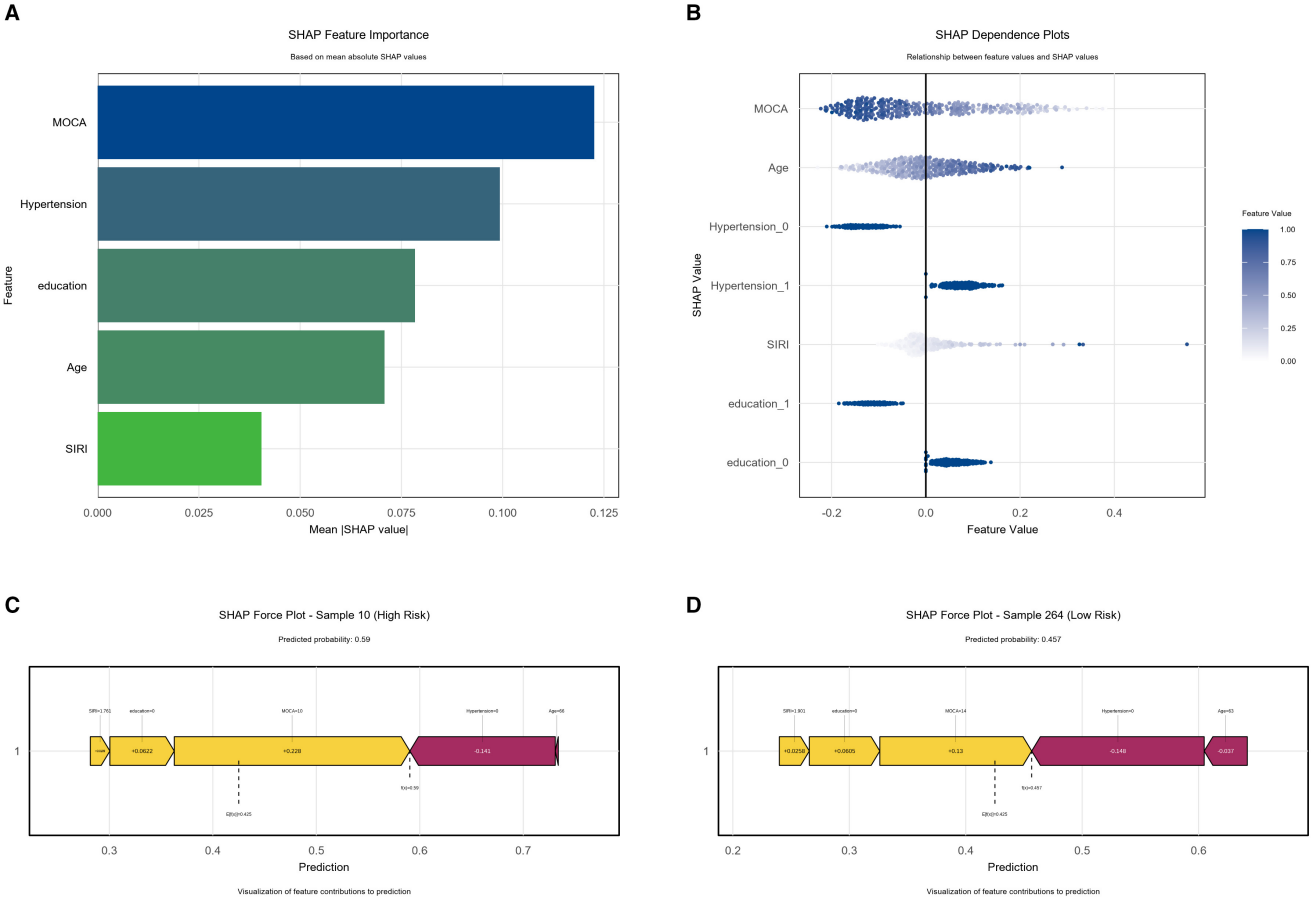
SHAP interpretation of model constructed by human–machine collaboration group. **(A)** The importance ranking of the model prediction features. The horizontal coordinate represents the SHAP values, the larger SHAP value indicates that the variable is more important; **(B)** each point represents a feature value, and different colors represent the final influence of the feature on the model output results, where deep color represents a larger value and light color represents a smaller value. **(C)** High-risk patient, **(D)** Decision-boundary patient.

SHAP-based logistic regression revealed feature importance ranking (descending order): MoCA score, age, hypertension, SIRI and education (Figure 6A). SHAP summary plots demonstrate how each feature positively or negatively predicts high BG-EPVS risk. In SHAP beeswarm plots, each point represents an individual's SHAP value, with color indicating feature magnitude (light color: low values, deep color: high values). The sum of individual SHAP values (higher values indicating greater probability of high BG-EPVS) is shown in Figure 6B. To demonstrate feature contributions, SHAP force plots illustrate individualized predictions for two representative cases. Yellow arrows indicate features negatively affecting prediction (decreasing SHAP values), while red arrows denote positive contributions (increasing SHAP values). Bar length represents contribution magnitude, with *E*[*f* (*x*)] denoting the base value (mean model prediction). In a high-risk BG-EPVS patient (Figure 6C), the model predicted probability of 0.590 exceeds the base value, indicating elevated risk. Figure 6D illustrates how BG-EPVS's risk factors affect the model's decision boundaries, while also visualizing the contribution of each feature.

## 4 Discussion

Our study identified age, hypertension, SIRI, MoCA, and education as independent risk factors for moderate to severe BG-EPVS. The study was confirmed that the SIRI is an independent risk factor for moderate-to-severe BG-EPVS. Calculated as (neutrophils × monocytes)/lymphocytes, SIRI represents a novel inflammatory biomarker that offers cost-effective and objective assessment of systemic inflammation and immune status (19, 20). Multivariate stepwise logistic regression revealed that SIRI surpassed traditional inflammatory markers—including NLR, SII, and neutrophil/monocyte counts—in predicting BG-EPVS, underscoring its potential for clinical predictive models.

### 4.1 Predictive superiority and mechanistic basis of SIRI

The advantage of SIRI lies in its integration of dynamic equilibrium data from multiple immune cell lineages (neutrophils, lymphocytes, monocytes), allowing more accurate reflection of neurovascular inflammation and immune responses (21–23). In contrast, traditional biomarkers such as NLR and SII rely on single parameters and may not fully capture the complexity of chronic inflammation. This study demonstrates significantly higher SIRI levels in patients with moderate-to-severe vs. mild BG-EPVS, confirming the central role of inflammation in EPVS pathogenesis. Chronic inflammation—a low-grade, non-infectious systemic inflammatory state—promotes EPVS through multiple pathways, including atherosclerosis, blood-brain barrier (BBB) disruption, and glymphatic dysfunction (24, 25). Specifically, inflammatory cells (e.g., neutrophils and monocytes) release diverse inflammatory factors and mediators (26), inducing endothelial dysfunction (27, 28) and compromising BBB integrity (19). Furthermore, microglial and macrophage activation triggers myelin loss (29), exacerbates BBB damage, promotes perivascular space fibrosis and occlusion, and ultimately impairs tissue fluid drainage, facilitating EPVS formation. These mechanisms are consistent with previous reports of SII and NLR associations with EPVS (15, 17), further validating our findings.

### 4.2 Clinical applications and risk stratification

Previous regional studies (30) have not identified a significant correlation between dNLR and EPVS, suggesting that geographical or population-specific variations may influence the applicability of certain inflammatory markers. Leveraging SIRI's predictive capability, we recommend its use as a complementary biomarker within existing evaluation frameworks rather than as a replacement for traditional indicators. SIRI enhances risk stratification by quantifying chronic inflammation linked to vascular health. High-risk individuals (predictive probability ≥45.7%) should receive prioritized MRI screening, whereas those at low risk (predictive probability < 45.7%) should undergo annual follow-up. This stratification approach optimizes resource allocation and enables personalized interventions, thereby improving EPVS severity prediction accuracy.

This study is the first to report a cross-sectional association between baseline SIRI and the severity of BG-EPVS. No significant correlation was observed between SIRI and centrum semiovale EPVS (CSO-EPVS). This finding suggests the potential involvement of small artery sclerosis mechanisms, wherein chronic inflammation may preferentially affect the more tortuous small arteries of the basal ganglia. We hypothesize that the temporal dynamics of SIRI may constitute a more predictive indicator. Serial measurements of SIRI could better reflect fluctuations in chronic systemic inflammation, which might be more directly linked to the chronic and progressive nature of EPVS pathology. Although the present cross-sectional design precludes the establishment of causal or temporal relationships, our data confirm a significant association between baseline SIRI levels and EPVS burden. Future longitudinal cohort studies incorporating SIRI trajectory measurements are warranted to elucidate its predictive value for cerebral microvascular disease progression. Furthermore, investigating the synergistic effects of SIRI with other inflammatory markers will provide a more comprehensive understanding of its role in cerebral microvascular pathophysiology. Future work should prioritize longitudinal data modeling and external validation to enhance the generalizability and clinical applicability of these findings.

### 4.3 Other relevant factors: cognitive performance and demographic variables

Beyond inflammatory markers, this study identified a significant correlation between cognitive performance and BG-EPVS burden. Each 1-point decrease in baseline MoCA score was associated with an 11% increase in BG-EPVS risk (*P* < 0.001), consistent with previous studies (31–33). EPVS may exacerbate cognitive impairment by impeding cerebrospinal fluid-interstitial fluid exchange and metabolic waste clearance (34). Greater EPVS severity reflects more pronounced neuropathological changes, including intracranial vascular inflammation and blood-brain barrier disruption.

Higher education level, a protective factor, may confer benefits through enhanced cognitive reserve and health literacy (35, 36). Individuals with more years of education are typically better equipped to access health-related information and implement effective coping strategies. Previous studies have demonstrated that a greater cognitive reserve is associated with a reduced risk of cognitive decline (37), suggesting that education plays a dual role by bolstering cognitive reserve and serving as a socioeconomic protective factor. Future longitudinal studies are needed to further elucidate the role of education in disease progression.

Multivariate logistic regression identified additional significant variables: age, smoking history, and hypertension. Age was the primary EPVS risk factor, with significantly older ages in moderate-to-severe cases, consistent with a meta-analysis of 8,395 participants (38). Hypertension showed significant association with BG-EPVS, while smoking and alcohol consumption history exerted indirect effects via atherosclerosis promotion (39).

### 4.4 Study limitations and future directions

This study has several limitations: its single-center design and small sample size may introduce selection bias; data relied solely on admission blood tests and brain MRI without longitudinal follow-up; and external validation was not performed. Future studies should employ larger-scale, multicenter prospective designs to collect baseline and longitudinal data, comprehensively evaluate SIRI's role in EPVS progression, and validate its clinical utility using imaging biomarkers.

## 5 Conclusions

We developed a predictive model integrating cognitive function and complete blood count-derived inflammatory biomarkers. This model quantifies the combined predictive value of SIRI and MoCA for BG-EPVS severity, highlights inflammation's multifaceted role in BG-EPVS pathophysiology, and underscores the clinical utility of these biomarkers for early detection and disease monitoring. These findings advance mechanistic understanding of EPVS pathogenesis and identify inflammatory pathways as potential therapeutic targets. Further studies should validate these observations and investigate longitudinal dynamics of inflammatory biomarkers in EPVS progression.

## Data Availability

The raw data supporting the conclusions of this article will be made available by the authors, without undue reservation.
